# Facile Fabrication of Co-Doped Porous Carbon from Coal Hydrogasification Semi-Coke for Efficient Microwave Absorption

**DOI:** 10.3390/molecules29194633

**Published:** 2024-09-29

**Authors:** Yan-Fang Li, Li-Fang Wang, Shu-Juan Gao, Tan-Lai Yu, Qi-Feng Li, Jun-Wei Wang

**Affiliations:** 1 Department of Chemical and Materials Engineering, Lyuliang University, Lvliang 033001, China; lllswlf@163.com (L.-F.W.); shujuangao@llu.edu.cn (S.-J.G.); 20171017@llu.edu.cn (T.-L.Y.); 2 Institute of New Carbon-Based Materials and Zero-Carbon and Negative-Carbon Technology, Lyuliang University, Lvliang 033001, China; 3 Institute of Coal Chemistry, Chinese Academy of Sciences, Taiyuan 033000, China; liqf@sxicc.ac.cn

**Keywords:** Co/C composites, microwave absorption, dielectric loss, magnetic loss

## Abstract

A Co-doped porous carbon was successfully fabricated by a facile carbonizing procedure using coal hydrogasification semi-coke (SC) as the carbon and cobalt nitrate as the magnetic precursors, respectively. The mass ratio of the precursors was changed to regulate the microwave absorption (MA) capabilities. The favorable MA capabilities are a result of a synergistic interaction be-tween the dielectric loss from the carbon framework, the magnetic loss from nano-sized Co particles, and multiple scattering from the residual pores. At a thickness of 4.0 mm, the Co/C composite showed the lowest reflection loss of −33.45 dB when the initial mass ratio of cobalt nitrate and SC was 1:1. The effective absorbing bandwidth (EAB) could achieve 3.5 GHz at 2 mm thickness. This work not only opens up a new avenue for the facile fabrication of dielectric and magnetic loss combinations and their structural design, but it also creates a new route for the high value-added exploitation of SC.

## 1. Introduction

With the increasing development of electronic and communication devices in recent years, more and more serious electromagnetic (EM) interference and pollution have caused great threats to the reliability of sensitive electronic devices, information security, and the health of human beings. Microwave absorbing materials (MAMs) are effective candidates to solve these issues by converting the incident EM wave to heat or other forms of energy and dissipating it [1,2,3,4].

In general, an ideal MAM should possess two significant characteristics: well-matched impedance and favorable attenuation constant. The better impedance matching means that more incident EM can enter the interior of an absorber and be dissipated, and the higher attenuation constant signifies stronger microwave dissipation capabilities. Hence, the purpose of high-performance absorbers is to improve impedance matching and increase microwave attenuation capabilities simultaneously. There are two main attenuation mechanisms, which include dielectric loss and magnetic loss [5,6]. Currently, carbon-based materials, as the main dielectric loss-type MAMs, have become ideal candidates due to their outstanding advantages of being lightweight, having high absorption efficiency, and having an adjustable microstructure. Typically, cost-effective, affordable, and resource-rich carbon-based MAMs, such as biomass-based or other carbonaceous residues, have attracted widespread attention [7,8,9]. Wu et al. prepared a variety of biomass-based absorbers using a one-step carbonization process. The obtained spinach-derived absorbers exhibited a maximum reflection loss (RL_min_) of −62.2 dB and a broad effective absorption bandwidth (EAB) of 7.3 GHz [10]. Our previous work used coal hydrogasification semi-coke (SC) and GO as the precursors to fabricate the absorbers by a calcination method. The composite exhibited good MA performance, with an EAB of 4.3 GHz and a RL_min_ of −48.8 dB at a thickness of 2.5 mm [11] whereas the carbon materials only endow the absorbers with dielectric loss, which is disadvantageous to the impedance matching. Previous reports have confirmed that an effective way to achieve favorable MA capabilities is to combine dielectric and magnetic loss materials (Fe, Co, Ni, and their oxides) [12,13]. This approach can not only utilize the benign magnetic loss capability of the magnetic components but also facilitate the practical applications limited by their high density [14,15,16].

There are multifold ways to combine the carbon materials and magnetic materials such as the sol–gel method [17], template strategy [18,19], metal organic framework (MOF) [20,21,22,23], carbon reduction treatment [24], the combination of these approaches, etc. [25]. For instance, Li et al. successfully prepared the tremella-like assemblies of hierarchically porous nickel cobalt/carbon (NiCo/C) by a microwave-assisted process and followed the sintering process. The obtained composite displayed a RL_min_ value of −41.6 dB with a mass ratio of 12.5% [26]. Wu et al. prepared Co/C crabapples via a solvothermal reaction coupled with a carbon reduction treatment. The composite exhibited a broad bandwidth of 5.9 GHz at an ultrathin thickness of 1.4 mm when the filling content was 50 wt% [27]. Fabrication of MOF is another efficient path for high-performance absorbers. They can be prepared through the reaction of metal ions and organic ligands via adding other high dielectric materials or not. Huang et al. prepared the MOF-based absorbers through a static reaction and heat treatment process using cobalt nitrate hexahydrate and 2-Methyl imidazole in the presence of luffa sponge carbon. The composites showed a RL_min_ of −60.81 dB and an EAB of 5.56 GHz at a very thin thickness of 1.68 mm [28]. Wang et al. fabricated a rod-like porous Co/C composite by directly carbonizing a Co-based MOF-74 precursor. The composite showed a RL_min_ of −38.46 dB with a coating thickness of 2.5 mm [29]. In a word, the preparation method should be directed toward simplicity, efficiency, and sustainability.

In this work, we selected SC and cobalt nitrate hexahydrate as the carbon and magnetic precursors. SC, as the residue of coal hydrogasification, exhibits several outstanding characteristics, such as high carbon content, preferable graphitization, special pore structure, and abundant sources [30]. SC has been widely applied in the fields of organic pollutant adsorbers [31], further hydrogasification for methane [32,33], and microwave absorbers [34,35]. Using SC as a carbon source can utilize its excellent dielectric properties, exhibits environmental friendliness, and allows for repurposing in the meanwhile. Using SC as the carbon precursor is part of this study’s uniqueness. A facile preparation method was adopted via a one-step calcination process, which can largely simplify the production process and shorten the time. The facile fabrication procedure is another advantage. The obtained Co/C composites inherited superb dielectric loss capability from the SC and magnetic loss from the Co particles. In addition, Co particles distributed on the walls and interior of the pores could greatly enhance the interfacial polarization. The 1:1 initial mass ratio of cobalt nitrate hexahydrate and SC showed the best microwave absorption performance with a RL_min_ of −33.45 dB and an EAB of 3.47 GHz at 2.0 mm thickness, making the porous Co/C composites a promising candidate for MAMs.

## 2. Results and Discussion

### 2.1. Morphology and Structure

**Figure 1:**
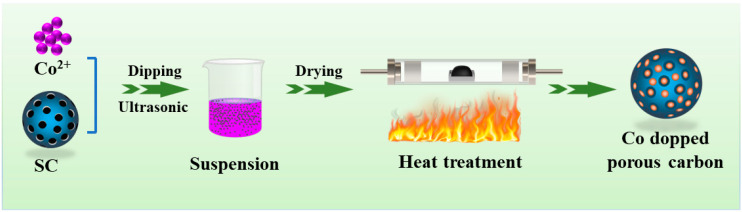
Schematic illustration of the synthesis process of Co-doped porous carbon composites.

Figure 1 shows the schematic synthesis process of Co-embedded porous carbon composites.

**Figure 2:**
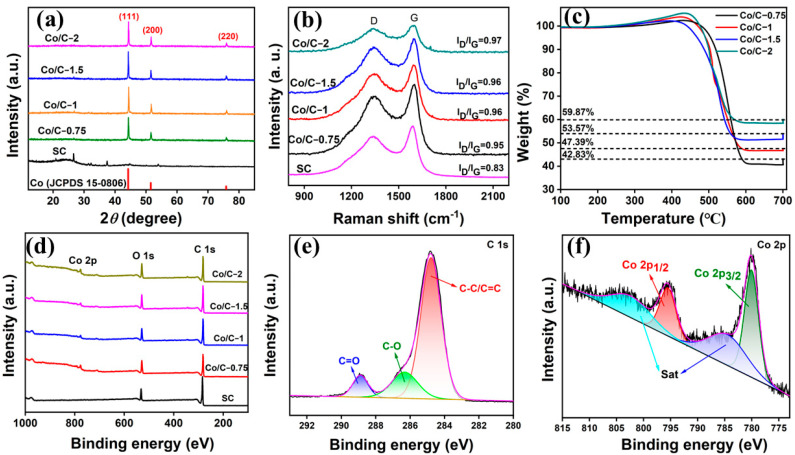
XRD patterns (**a**) and Raman spectra (**b**) of SC and Co/C composites; TGA curves for Co/C composites (**c**). XPS survey spectra of the samples (**d**) and the high-resolution spectrum of C1s (**e**) and Co2p (**f**) for Co/C−1.

The X-ray diffraction technique was used to verify the crystalline and phase composition of SC and Co/C composites. As shown in Figure 2a, different proportions of precursors have no obvious influence on the composition of Co/C composites, reflecting nearly the same peaks on the patterns. Specifically, all the Co/C samples have three strong diffraction peaks located at 44.3°, 51.6°, and 75.9°, which can be indexed to the (111), (200), and (220) planes of face-centered cubic cobalt (JCPDS No. 15-0806), respectively [36]. The results confirm the in situ reduction of Co^2+^ to Co metal during the calcination process. The sharp peak of SC pattern located at 26.6° responds to the graphitized crystal plane (002) of carbon, indicating that SC has certain graphitized carbon domains. On the other hand, the diffraction peak of Co/C composites at 26.6° becomes much fainter compared with SC, which is direct evidence of the reaction between SC and cobalt nitrate hexahydrate in the pyrolysis process. Particularly, there also appear other peaks, except for at 26.6° and 44.6°. This is consistent with our previous research results that demonstrates that SC not only contains C but also includes other elements such as O, N, Fe, Mn, Ni, etc. These components are the source of other peaks [11]. The existence of these elements also contributes to dipole and interface polarization, which is conducive to the enhancement of microwave absorption.

Raman spectroscopy was further used to investigate the effect of cobalt salt on the carbon graphitization degree during the carbonization process, as illustrated in Figure 2b. There are two characteristic peaks in each sample, which are roughly at 1350 cm^−1^ (D-band) and 1580 cm^−1^ (G-band). The local defect and the disordered carbon are represented by the D-band, whereas the graphited C atoms observed for sp^2^ carbon domains are represented by the G-band. The peak intensity of D-band to G-band (I_D_/I_G_) is usually used to evaluate the degree of graphitization of carbon-based materials [37,38]. As shown in Figure 2b, the Co/C composites exhibit a much higher I_D_/I_G_ than that for SC (0.83). Since cobalt salt could catalyze the graphitization of carbon, the increased I_D_/I_G_ value could be attributed to the transitional stage from amorphous carbon to nanocrystalline graphite according to the phenomenological three-stage model proposed by Ferrari and Roberston [39]. Due to the nano size of crystalline graphite, the I_D_/I_G_ values show a declining tendency. In addition, the higher the content of the cobalt salt precursor, the higher the I_D_/I_G_ value. Specifically, the I_D_/I_G_ value rises from 0.95 to 0.97 for Co/C−0.75 and Co/C−2. The enhancement of nanocrystalline graphite would promote conductivity and polarization capability, which favors conductive loss and polarization loss for better microwave absorption.

Thermogravimetric analysis (TGA) was carried out to determine the accurate content of Co nanoparticles in Co/SC composites. Figure 2c displays the thermogravimetric curves of Co/C composites in an air atmosphere. The samples were heated to 100 °C and kept for 30 min for the removal of adsorbed water. The samples were kept for 4 h at 700 °C for the sufficient redox reaction. The weight fluctuation of the samples mainly originated from the oxidation of Co nanoparticles to Co_3_O_4_, the combustion of carbon to CO_2_ or other carbon oxides, and the splitting of other components such as O and N. The weight increase from 100 °C to ~440 °C is mainly attributed to the oxidation of Co to Co_3_O_4_ while the sharp decline from ~440 °C to ~580 °C is primarily ascribed to carbon consumption. The weight percentage was finally constant at 42.83%, 47.39%, 53.57%, and 59.87%, respectively. The content of Co in the Co/C composites can be evaluated via the following formula:

(1) $$\mathrm{wt}\%(\mathrm{Co})=\frac{3\times M(\mathrm{Co})\times R}{M({\mathrm{Co}}_{3}O_{4})}$$

where wt%(Co) represents the content of Co in the Co/C composites, and *R* stands for the residual weight percentage after the reaction. *M*(Co) and *M*(Co_3_O_4_) represent the molecular weights of Co and Co_3_O_4_, respectively [40]. Hence, the contents of Co nanoparticles in Co/C−0.75, Co/C−1, Co/C−1.5, and Co/C−2 composites were determined to be 31.48 wt%, 34.83 wt%, 39.37 wt%, and 44.00 wt%, respectively. The above results illustrate that the Co content in Co/C composites has positive correlation with the doping of cobalt salt.

The chemical state information on the surface of the samples was characterized by XPS. Figure 2d shows the survey spectra of SC and Co/C composites. The Co/C composites show three typical peaks located at 282 eV, 529 eV, and 776 eV, corresponding to C 1s, O 1s, and Co 2p, respectively, which demonstrate the existence of C, O, and Co elements in Co/C composites. The high-resolution C 1s XPS spectrum (Figure 2e) of Co/C−1 consists of three deconvoluted peaks at 284.8 eV, 286.4 eV, and 288.9 eV, corresponding to C–C/C=C, C–O, and C=C functional groups, respectively. Four different Co species types are visible in the high-resolution Co 2p XPS spectra. The peaks at 780.8 eV and 795.6 eV correspond to Co 2p_3/2_ and Co 2p_1/2_, respectively, while the peaks at 784.8 eV and 803.3 eV are the satellite peaks of Co 2p from Co metal. The results are consistent with the analysis of XRD that Co metal is the main existence state [41].

**Figure 3:**
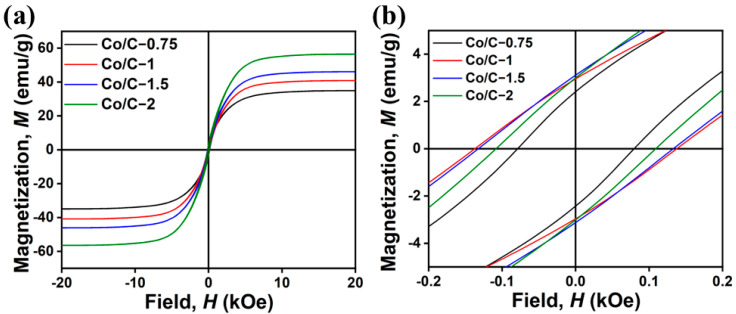
VSM hysteresis loop (**a**) and the magnified image of Co/C composites (**b**).

The magnetic properties of all the samples were measured at 300 K. As shown in Figure 3, all the Co/C composites exhibit typical ferromagnetic characteristics with magnetic hysteresis loops. Co/C composites have considerably superior magnetic characteristics than those of SC (Figure S1), suggesting that magnetic elements were successfully incorporated during the carbonization process. The saturation magnetization (*M*s) values of the Co/C composite increase with elevated Co doping, which is consistent with the TGA results for increased Co content from Co/C−0.75 to Co/C−2. Specifically, the *M*s values of Co/C−0.75, Co/C−1, Co/C−1.5, and Co/C−2 are 34.93 emu/g, 40.83 emu/g, 45.78 emu/g, and 56.44 emu/g, respectively. This is attributed to increasingly growing Co grains and the reduction of lattice defects with more Co doping. Of note, Co/C−1 displays the highest coercive forces (*H*_c_, 138 Oe) compared to those of other Co/C composites. The extraordinary *H*_c_ value of Co/C−1 is mainly related to the higher magnetocrystalline anisotropy of Co nanoparticles, which is shown in SEM images as dynamic shapes and wider size distribution. The results demonstrate that these Co/C composites with various morphologies and dimensions possess tailorable intrinsic dielectric properties and magnetic response abilities.

**Figure 4:**
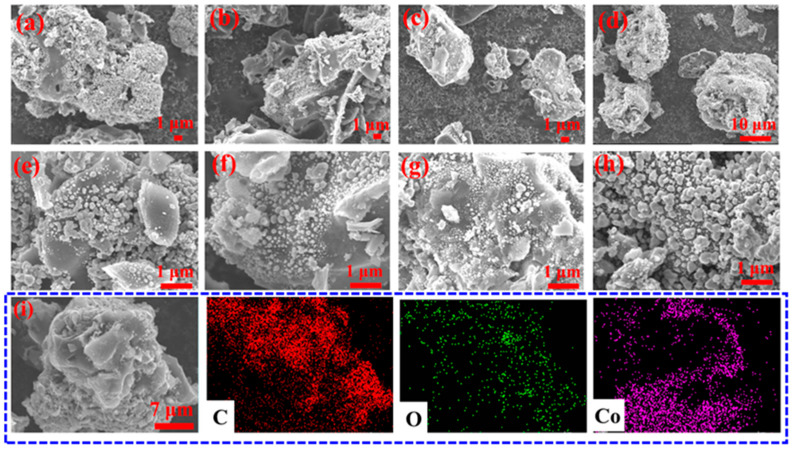
The SEM images of the Co/C−0.75 (**a**,**e**), Co/C−1 (**b**,**f**), Co/C−1.5 (**c**,**g**), and Co/C−2 (**d**,**h**). The element mapping analysis of Co/C−1 (**i**).

The morphologies and structures were characterized by SEM, as shown in Figure 4. The composites inherited plenty of pores from SC (Figure S2). Abundant spherical, quasi-spherical and irregular Co particles are distributed in the pores and walls of the SC, resulting in a sharp decrease in the pores compared with SC. Different levels of Co doping have a significant effect on the size and distribution of Co nanoparticles. The size and distribution of Co particles were measured via the nano measurer software v1.2.5 from the SEM images. Co/C−2 possesses the biggest Co mean size of ~150 nm and the widest distribution. In contrast, the mean sizes of Co/C−1 and Co/C−1.5 are relatively small at ~100 nm and ~60 nm, respectively. Remarkably, Co/C−1 shows a wider distribution than other Co/C composites, combined with its dynamic spherical, quasi-spherical, and irregular shapes, contributing to a better shape anisotropy. The results illustrate that the precursors participate in the reaction process, thus influencing the growth of grains, dynamic sizes, and distributions. According to our previous analysis, possible reactions in the annealing process are listed as follows:

$$Co{\left({NO}_{3}\right)}_{3}+C\to Co+CO_{2}+NO_{2}$$

$$Co{\left({NO}_{3}\right)}_{3}\to {Co}_{3}O_{4}+{NO}_{2}+O_{2}$$

$${C+O}_{2}\to {\mathrm{CO}}_{2}$$

$${\mathrm{CO}}_{2}+C\to 2\mathrm{CO}$$

$${\mathrm{Co}}_{3}O_{4}+C\;\;(\mathrm{or}\;\;\mathrm{CO})\to \mathrm{Co}+{\mathrm{CO}}_{2}$$

The EDS mapping of Co/C−1 composites was performed to observe the elemental distribution. As shown in Figure 4i, Co, O, and C atoms uniformly distribute in the composites, indicating that the Co nanoparticles embedded in porous carbon composites were successfully fabricated.

### 2.2. Microwave Absorption Performance

**Figure 5:**
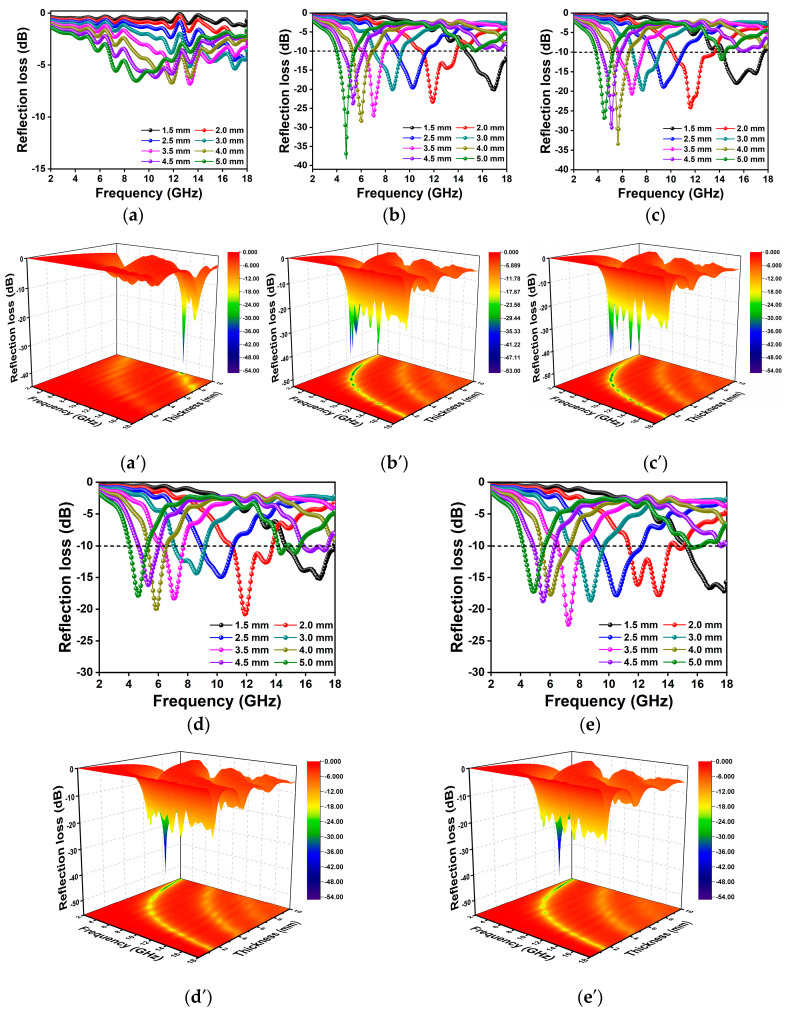
Calculated RL curves and 3D plots of SC (**a**,**a′**), Co/C−0.75 (**b**,**b′**), Co/C−1 (**c**,**c′**), Co/C−1.5 (**d**,**d′**), and Co/C−2 (**e**,**e′**).

The EM absorption performance of the as-prepared composites was studied through the calculated RL value according to the following equations:

(2) $$\mathrm{RL}(\mathrm{dB})=20\log\left|\frac{Z_{\mathrm{in}}-Z_{0}}{Z_{i}+Z_{0}}\right|$$

(3) $$Z_{\mathrm{in}}=Z_{0}{\left(\mu_{r}/\varepsilon_{r}\right)}^{0.5}\tanh\left(2\pi \mathrm{jfd}/c\sqrt{\mu_{r}\varepsilon_{r}}\right)$$

where $Z_{0}$ is the air impedance, $Z_{\mathrm{in}}$ is the input impedance, *d* is the thickness of the absorbers, *f* represents the microwave frequency, and *c* is the velocity of light in free space. $\varepsilon_{r}$ and $\mu_{r}$ stand for complex permittivity and complex permeability, respectively. The RL value below −10 dB is regarded as an effective microwave absorption, which means more than 90% of the electromagnetic energy can be attenuated, and the corresponding frequency range is called the effective absorption bandwidth (EAB). The RL curves and the 3D plots of SC and Co/C composites in the range of 2–18 GHz are displayed in Figure 5. As shown in Figure 5, all the Co/C composites show an enhanced MA compared with SC, demonstrating that Co incorporation has a significant effect on composition, structure, and electromagnetic parameters, which endows the composites with improved MA performance. The RL_min_ of SC is far below −10 dB at the calculated matching thicknesses. Although Co/C−0.75 has the strongest absorbing ability with a RL_min_ value of −38.33 dB, the matching thickness is as high as 5.0 mm, which is disadvantageous for practical applications. Co/C−1 exhibits a moderate RL_min_ of −33.45 dB at a thickness of 4.0 mm. The EAB of Co/C−1 is 3.47 GHz (10.15–13.62 GHz) at 2.0 mm thickness. The RL_min_ of Co/C−1.5 and Co/C−2 show a declining tendency compared with that of Co/C−1, which indicates that different compositions, sizes, and microstructures of Co/C composites influence electromagnetic parameters, thus leading to diverse MA performances.

**Figure 6:**
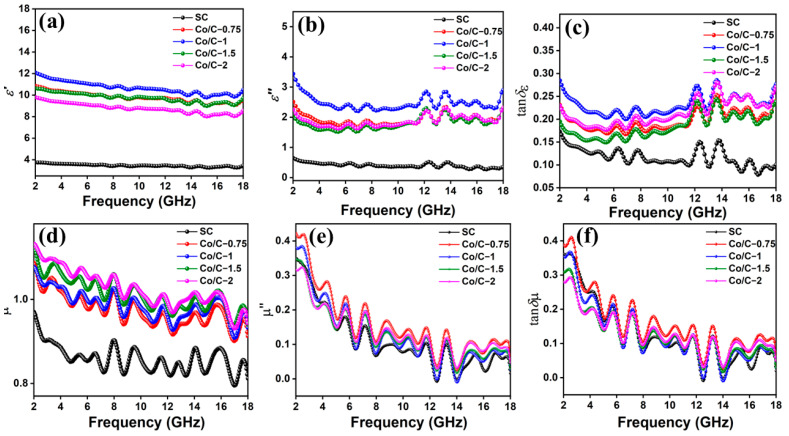
Real (**a**) and imaginary (**b**) permittivity, real (**c**) and imaginary (**d**) permeability, dielectric (**e**) and magnetic (**f**) loss tangent of the composites.

The complex permittivity (ε_r_ = ε′ − jε″) and permeability (μ_r_ = μ′ − jμ″) are the primary determinants for the EM properties. ε′ and μ′ denote the ability to store electric and magnetic energies, respectively, while ε″ and μ″ represent the ability to dissipate the electric and magnetic energies, respectively. To further investigate the relationship between the complex permittivity, complex permeability, and MA performances, the frequency dependences of permittivity and permeability are depicted, as shown in Figure 6. In Figure 6a, all the samples display a downward tendency with the increased frequency, which can be attributed to the dielectric polarization relaxation behavior of Co/C−paraffin hybrids. The ε′ and ε′ values of SC fluctuate from 3.74 to 3.45 and 0.65 to 0.35, respectively, which are much lower than those of Co/C composites. This is attributed to the fact that SC has an inferior electric field storage capability, indicating that its electrical conductivity and graphitization are not satisfactory. The electric storage and dissipation abilities are attributed to carbon and Co for Co/C composites. Among the Co/C composites, Co/C−1 exhibits outstanding electric storage and attenuation capabilities, which is mainly ascribed to its peculiar microstructure. Although the graphitization degree of Co/C−1 is moderate, the variform Co nanoparticles and the resulting abundant pores made a more prominent contribution to the conductivity and polarization capabilities than its counterparts, bringing about higher ε′ (12.08–10.49) and ε″ (3.44–2.90). On the other hand, more and bigger Co particles restrict the polarization of dipoles for better electric storage capability; thus, Co/C−2 exhibits a decrease. The incorporation of magnetic Co nanoparticles greatly enhanced the magnetic storage and attenuation capabilities, showing much higher μ′ and μ″ for the Co/C composites compared with SC. Of note, Co/C−1 exhibits the lowest μ′ and μ″; this could be ascribed to its highest *H*_c_, as shown in the previous VSM analysis. A high *H*_c_ means more difficult magnetizability for magnetic energy storage and dissipation capabilities, resulting in the relatively low μ′ and μ″ for Co/C−1. In addition, the dielectric loss and magnetic loss tangents (tanδ_ε_ = ε″/ε′, tanδ_µ_ = μ″/μ′) are crucial parameters to determine the dominant loss mechanism in the MA mechanism. As shown in Figure 6c,f, dielectric loss is the main contribution to the MA performance of Co/C−1 because the tanδε values (above 0.24) are larger than the tanδμ values (below 0.2) in almost all the frequency ranges, demonstrating that dielectric loss is the dominant loss mechanism for the attenuation of electromagnetic waves [41].

To further investigate the existence of dielectric polarization and the related relaxation, the relationship between ε′ and ε″ was analyzed based on the Debye relaxation theory:

(4) $${\left(\varepsilon^{\prime }-\frac{\varepsilon_{s}+\varepsilon_{\infty }}{2}\right)}^{2}+{\left(\varepsilon^{\prime \prime }\right)}^{2}={\left(\frac{\varepsilon_{s}-\varepsilon_{\infty }}{2}\right)}^{2}$$

**Figure 7:**
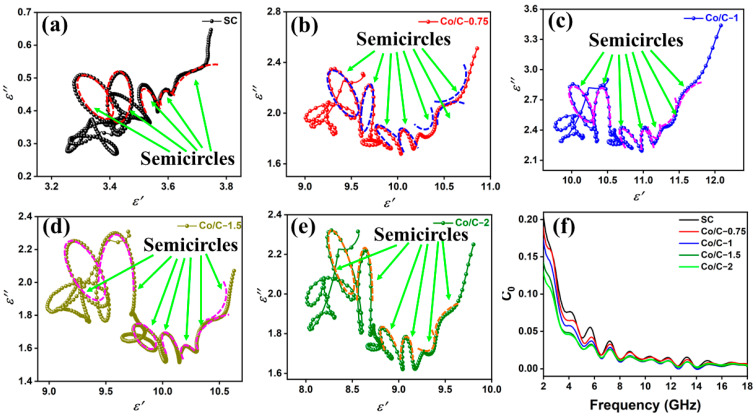
Cole–Cole diagrams for the paraffin composites (**a**–**e**) and the the *C*_0_ − *f* curves of the composites (**f**).

In the curve of ε′~ε″, an approximate semicircle (Cole–Cole semicircle) represents one Debye relaxation process. The polarization in the tested frequency range mainly includes interfacial polarization and dipolar polarization. The Cole–Cole plots are shown in Figure 7. As shown in Figure 7a–e, Co/C composites exhibit more semicircles compared to SC, which is attributed to the introduction of multiple nano-heterointerfaces due to the incorporation of Co nanoparticles and Co embedding into the pores of SC. Specifically, SC shows five distinct semicircles, while the Co/C composites exhibit seven distinct semicircles, with the exception of Co/C−2. Remarkably, Co/C−2 exhibits six distinct semicircles and a longer tail than other Co/C composites. The tail is the symbol of conductive loss, suggesting that Co/C−2 possesses stronger conductive ability [42].

The magnetic loss mechanism was analyzed. The eddy current loss and natural resonance are the dominant magnetic loss mechanisms in the GHz range. The following formula is usually used to analyze the eddy current effect on EM absorbing performance:

(5) $$C_{0}=\mu^{\prime \prime }{\left(\mu^{\prime }\right)}^{-2}f^{-1}$$

If *C*_0_ is constant in the curve of *C*_0_ − *f*, the eddy current loss is considerable. As shown in Figure 7f, $C_{0}$ is relatively constant in the high-frequency range of 16–18 GHz. Therefore, eddy current loss is the dominant magnetic loss mechanism in 16–18 GHz. There are multiple fluctuations in the rest of the frequency range of 2–16 GHz, which is evidence of the coexistence of eddy current loss and natural resonance. The fluctuations in the curve of *C*_0_ − *f* are consistent with those of $\mu^{\prime \prime }$, which is attributed to the magnetic components of SC and nano-sized Co particles. For one thing, different magnetic components of SC and incorporated Co correspond to different resonance frequencies, exhibiting different resonance peaks. For another thing, the synergistic effect of the nanometer size effect as well as the surface effect of Co lead to multiple fluctuations [43].

**Figure 8:**
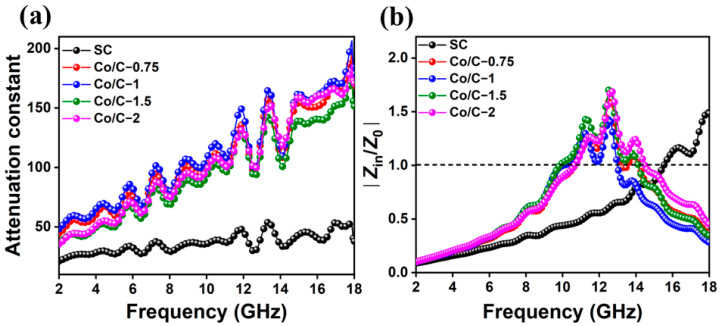
The attenuation constant (**a**) and impedance matching at 2.0 mm thickness (**b**) curves of the composites.

To further investigate the influence of impedance matching and microwave attenuation capability on MA performances, the calculated attenuation constant (α) and impedance matching are shown in Figure 8. The higher α value means stronger attenuation capability of an absorber to dissipate EM energies. If the input impedance of an absorber is equal to that of the free space, the value of |*Z*_in_/*Z*_0_| is 1. A |*Z*_in_/*Z*_0_| value closer to 1 means more input EM waves enter the interior of an absorber and are dissipated. A high α and benign impedance matching capability are two prominent factors for favorable MA performances. As shown in Figure 8, SC exhibits the lowest α compared with Co/C composites, indicating the weakest EM attenuation capability; meanwhile, the value of |*Z*_in_/*Z*_0_| is far less than 1, which manifests the inferior impedance matching performance. Although the impedance matching of the Co/C composites show little difference, Co/C−1 exhibits the highest α, indicating the strongest MA attenuation capabilities [44]. The results are consistent with the calculated RL results.

### 2.3. Microwave Absorption Mechanism

**Figure 9:**
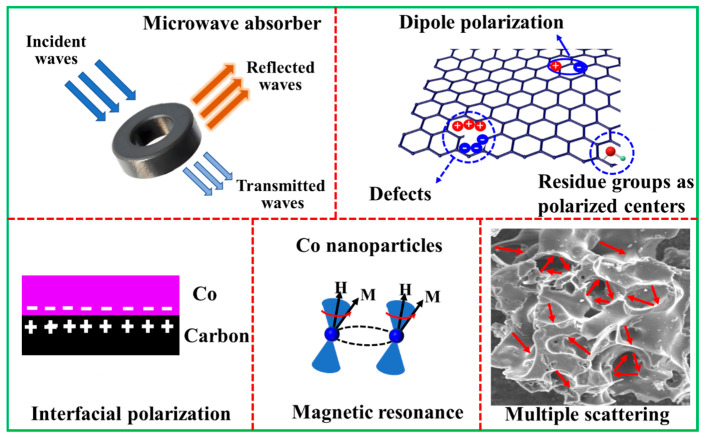
Schematic illustration of the microwave absorption mechanism.

The microwave absorption mechanism of the Co/C composites is illustrated in Figure 9. First, the composites not only inherited several defects and residual groups (C=O, C–O) from SC, but they also received many defects due to the presence of Co, which acts as the polarization center and contributes to dipole polarization loss. The fabrication process of the composites also brought about plentiful heterogeneous interfaces (Co/C, Co/air, and C/air), which is conducive to interfacial polarization loss for favorable MA performances. Secondly, the graphited carbon and Co nanoparticles provide suitable local conductivity, which brings high conductive loss. Third, because of the significant shape anisotropy, the nano-sized Co in the composites increases the magnetic losses by promoting the formation of exchange resonance and domain-wall resonance in magnetic entities. In addition, the residual pores from SC could prolong the EM wave transmission path in the composites through multiple scattering, which is beneficial for the conversion of EM wave energy to other sorts of energies. The pores are also helpful for impedance matching. The synergistic effects of the above-mentioned factors jointly contribute to overall high-performance MA.

## 3. Materials and Methods

### 3.1. Materials

SC, acquired from the Institute of Coal Chemistry, was pulverized and passed through a 140-mesh screen. Cobalt nitrate hexahydrate (Co(NO_3_)_2_·6H_2_O) was obtained from Shanghai Aladdin biochemical technology Co., Ltd (Shanghai, China), Deionized water (DI) was homemade in our lab. SC was dried at 80 °C before use.

### 3.2. Preparation of Hierarchical Porous Co/C Absorbers

Co-embedded porous carbon was prepared via facile impregnation coupled with a calcination strategy. After dissolving Co(NO_3_)_2_·6H_2_O in deionized water, a certain amount of SC was added to the solution of Co(NO_3_)_2_·6H_2_O. The suspension was sonicated for 30 min and kept overnight. The mixture was then stirred through a magnetic heated stirrer and dried in a vacuum oven until the solvent was completely evaporated. The absorbers were prepared by calcinating the above precursor hybrids at 700 °C for 2 h in a N_2_ atmosphere. The absorbers with different initial Co(NO_3_)_2_·6H_2_O and SC mass ratios of 0.75:1, 1:1, 1.5:1, and 2:1 were marked as Co/C−0.75, Co/C−1, Co/C−1.5, and Co/C−2, respectively.

### 3.3. Characterization

X-ray powder diffraction (XRD; Advance D8, Bruker, Billerica, USA) was used to characterize the crystallization of the absorbers utilizing Cu Kα (λ = 1.5406 Å) at 40 mA and 40 kV in the 2θ range of 10°–90°. The surface morphologies and elemental mapping distributions were examined using scanning electron microscopy (SEM; JSM-7900F, JEOL, Tokyo, Japan) and energy dispersive spectroscopy (EDS; X Flash 5010, Bruker, Billerica, USA), respectively. Using a 532 nm laser beam as the light source, Raman spectroscopy (Raman; Dxr2xi, Thermo Fisher Scientific, Waltham, MA, USA) was used to record Raman spectra. Utilizing Al Kα (1486.6 eV) as the monochromatic source, X-ray photoelectron spectroscopy (XPS; ESCALAB 250Xi, Thermo Fisher Scientific, Waltham, MA, USA) was used to describe the chemical states of the produced hybrids. The magnetic hysteresis loops were measured using a vibrating sample magnetometer (VSM; 7404, Lake Shore Cryotronics, Westerville, OH, USA) at room temperature. The thermogravimetric analysis was carried out using a thermal analyzer (TGA; TGA-50, Shimadzu Scientific Instruments, Kyoto, Japan) in an air atmosphere. The samples were heated to 100 °C with a heating rate of 10 °C/min and kept for 30 min, and they were then heated continually to 700 °C and kept for 4 h.

The coaxial-line approach was utilized to assess the complex permittivity and permeability between 2 GHz and 18 GHz with a vector network analyzer (VNA; E5071C, E5071C, Agilent Technologies, Santa Clara, CA, USA). The measured composites and paraffin were uniformly mixed in a mass ratio of 6:4 and shaped into a ring with a toroidal shape ($\Phi_{\mathrm{out}}$: 7.00 mm, $\Phi_{\mathrm{in}}$: 3.04 mm). According to the transmission theory, the reflection loss, the attenuation constant, and the impedance matching were determined.

## 4. Conclusions

In summary, a magnetic Co-embedded heterostructure in coal-derived carbon framework was successfully prepared by a facile and cost-effective thermal annealing method. The composition, morphology, size, and graphitization were adjusted via the initial mass ratio of the carbon and magnetic precursors. Graphited carbon, nano-sized Co, residual groups, and their synergistic effects on dipole and interfacial polarization, conductivity loss, magnetic loss, and multiple scattering lead to excellent MA performances. The results show that when the thickness is 4.0 mm, the RL_min_ is as low as −33.45 dB, and the effective absorption bandwidth is 3.47 GHz at 2.0 mm thickness. This work provides a facile strategy for the fabrication of highly efficient absorbers and opens up a new path for the high value-added utilization of SC. In contrast, the composites with paraffin as the matrix are somewhat environmental instable. This could be solved by using a polymer as the matrix through solution mixing or direct blending. The focus of future research should be on high-performance, cost-effective, and practical multi-functional applications.

## Data Availability

Data are contained within the article and Supplementary Materials.

## Supplementary Materials

The following supporting information can be downloaded at https://www.mdpi.com/article/10.3390/molecules29194633/s1: Figure S1: The SEM images of SC.; Figure S2: Magnetic hysteresis loops of SC.

## References

[1] Guan H., Wang Q., Wu X., Pang J., Jiang Z., Chen G., Dong C., Wang L., Gong C. (2021). Biomass derived porous carbon (BPC) and their composites as lightweight and efficient microwave absorption materials. Compos. Part B Eng..

[2] Lv H., Yang Z., Pan H., Wu R. (2022). Electromagnetic absorption materials: Current progress and new frontiers. Prog. Mater. Sci..

[3] Zhao H., Cheng Y., Liu W., Yang L., Zhang B., Wang L.P., Ji G., Xu Z.J. (2019). Biomass-Derived Porous Carbon-Based Nanostructures for Microwave Absorption. Nano-Micro Lett..

[4] Zhou M., Gu W., Wang G., Zheng J., Pei C., Fan F., Ji G. (2020). Sustainable wood-based composites for microwave absorption and electromagnetic interference shielding. J. Mater. Chem. A.

[5] Li W., Liu Y., Guo F., Du Y., Chen Y. (2021). Self-assembly sandwich-like Fe, Co, or Ni nanoparticles/reduced graphene oxide composites with excellent microwave absorption performance. Appl. Surf. Sci..

[6] Qu Z., Wang Y., Wang W., Yu D. (2021). Robust magnetic and electromagnetic wave absorption performance of reduced graphene oxide loaded magnetic metal nanoparticle composites. Adv. Powder Technol..

[7] Lu X., Li X., Zhu W., Xu H. (2022). Construction of embedded heterostructures in biomass-derived carbon frameworks for enhancing electromagnetic wave absorption. Carbon.

[8] Yang W., Yan L., Jiang B., Wang P., Li Z., Wang C., Bai H., Zhang C., Li Y. (2022). Crumpled nitrogen-doped porous carbon nanosheets derived from petroleum pitch for high-performance and flexible electromagnetic wave absorption. Ind. Eng. Chem. Res..

[9] Wang H., Meng F., Li J., Li T., Chen Z., Luo H., Zhou Z. (2018). Carbonized Design of Hierarchical Porous Carbon/Fe_3_O_4_@Fe Derived from Loofah Sponge to Achieve Tunable High-Performance Microwave Absorption. ACS Sustain. Chem. Eng..

[10] Wu Z., Tian K., Huang T., Hu W., Xie F., Wang J., Su M., Li L. (2018). Hierarchically porous carbons derived from biomasses with excellent microwave absorption performance. ACS Appl. Mater. Interfaces.

[11] Li Y., Li X., Li Q., Zhang R., Zhao Y., Wang J. (2022). Fabrication of flexible thermoplastic polyurethane/coal hydrogasification semi-coke composites with low rGO content for high-performance microwave absorption. ACS Appl. Electron. Mater..

[12] Zhang D., He W., Quan G., Wang Y., Su Y., Lei L., Du Y., Hong Y., Wang S., Tang Y. (2023). Sterculia lychnophora seed-derived porous carbon@CoFe_2_O_4_ composites with efficient microwave absorption performance. Appl. Surf. Sci..

[13] Zhou C., Wang X., Luo H., Deng L., Wang S., Wei S., Zheng Y., Jia Q., Liu J. (2019). Interfacial design of sandwich-like CoFe@Ti_3_C_2_T_x_ composites as high efficient microwave absorption materials. Appl. Surf. Sci..

[14] Wang Y., Zhao P., Liang B., Wan G.-S. (2023). Carbon nanotubes decorated Co/C from ZIF-67/melamine as high efficient microwave absorbing material. Carbon.

[15] Quan B., Xu G., Gu W., Sheng J., Ji G. (2019). Cobalt nanoparticles embedded nitrogen-doped porous graphitized carbon composites with enhanced microwave absorption performance. J. Colloid Interface Sci..

[16] Zhou X., Zhao B., Lv H. (2023). Low-dimensional cobalt doped carbon composite towards wideband electromagnetic dissipation. Nano Res..

[17] Liu Q., Zhang D., Fan T. (2008). Electromagnetic wave absorption properties of porous carbon/Co nanocomposites. Appl. Phys. Lett..

[18] Yan J., Huang Y., Chen C., Liu X., Liu H. (2019). The 3D CoNi alloy particles embedded in N-doped porous carbon foams for high-performance microwave absorbers. Carbon.

[19] Wang L., Du Z., Bai X., Lin Y. (2021). Constructing macroporous C/Co composites with tunable interfacial polarization toward ultra-broadband microwave absorption. J. Colloid Interface Sci..

[20] Wu Q., Wang J., Jin H., Dong Y. (2020). Facile synthesis of Co-embedded porous spherical carbon composites derived from Co_3_O_4_/ZIF-8 compounds for broadband microwave absorption. Compos. Sci. Technol..

[21] Zhang Y., Zhang H.-B., Wu X., Deng Z., Zhou E., Yu Z.-Z. (2019). Nanolayered cobalt@carbon hybrids derived from metal–organic frameworks for microwave absorption. ACS Appl. Nano Mater..

[22] Jin H., Wang J., Yang S., Wu Q., Zhang B. (2021). ZIF-67-derived micron-sized cobalt-doped porous carbon-based microwave absorbers with g-C_3_N_4_ as template. Ceram. Int..

[23] Sun Q., Li J., Zhang H., He X., Wu B., Wang J., Mahmood N., Jian X. (2024). The MOFs/COFs-derivant decorating FeSiAl coupling magnetic and electrical losses for enhanced microwave absorption. Appl. Surf. Sci..

[24] Liang J., Chen J., Shen H., Hu K., Zhao B., Kong J. (2021). Hollow porous bowl-like nitrogen-doped cobalt/carbon nanocomposites with enhanced electromagnetic wave absorption. Chem. Mater..

[25] Fu M., Yu H., Chen W. (2023). Construction of Co_3_O_4_ porous rod/graphene heterostructures toward strong and broadband microwave absorption applications. Appl. Surf. Sci..

[26] Li C., Sui J., Zhang Z., Jiang X., Zhang Z., Yu L. (2019). Microwave-assisted synthesis of tremella-like NiCo/C composites for efficient broadband electromagnetic wave absorption at 2–40 GHz. Chem. Eng. J..

[27] Wu N., Liu C., Xu D., Liu J., Liu W., Liu H., Zhang J., Xie W., Guo Z. (2019). Ultrathin high-performance electromagnetic wave absorbers with facilely fabricated hierarchical porous Co/C crabapples. J. Mater. Chem. C.

[28] Huang Y., Tian K., Zhang C., Wang J., Shu R., Chen Z., Liu X., Li Y., Xu L. (2023). Lightweight and efficient luffa sponge carbon/Co composites with adjustable electromagnetic wave absorption properties. J. Colloid Interface Sci..

[29] Wang X., Guan Y., Zhang R., Zhou P., Song Z., Wang M., Wang L., Zhang Q. (2021). Facile synthesis of cobalt nanoparticles embedded in a rod-like porous carbon matrix with excellent electromagnetic wave absorption performance. Ceram. Int..

[30] Li Y., Li X., Kang M., Zhao Y., Li Q., Wang J. (2023). Fabrication of flexible waterborne polyurethane/Fe-doped residual carbon from coal hydrogasification semi-coke composites for high-performance microwave absorption. Compos. Part A Appl. Sci. Manuf..

[31] Li P., Ailijiang N., Cao X., Lei T., Liang P., Zhang X., Huang X., Teng J. (2015). Pretreatment of coal gasification wastewater by adsorption using activated carbons and activated coke. Colloids Surf. A Physicochem. Eng. Asp..

[32] Ding X., Zhang Y., Zhang T., Tang J., Xu Y., Zhang J. (2013). Effect of Operational Variables on the Hydrogasification of Inner Mongolian Lignite Semicoke. Energy Fuels.

[33] Ding X.K., Zhang T.K., Zhang Y.F., Xu Y., Zhang G.J. (2013). Hydrogasification of Low-Oxygen Semi-Coke to Produce Methane by Consuming Less Hydrogen. Appl. Mech. Mater..

[34] Gao F., Li Y., Mao L., Wang Y., Zhu B., Liang L., Li G., Zhang R. (2022). Facile synthesis of Co/SC microwave absorbents by recycling coal hydrogasification residue. Mater. Lett..

[35] Gao S., Chen L., Zhang Y., Shan J. (2021). Fe nanoparticles decorated in residual carbon from coal gasification fine slag as an ultra-thin wideband microwave absorber. Compos. Sci. Technol..

[36] Deng J., Li S., Zhou Y., Liang L., Zhao B., Zhang X., Zhang R. (2018). Enhancing the microwave absorption properties of amorphous CoO nanosheet-coated Co (hexagonal and cubic phases) through interfacial polarizations. J. Colloid Interface Sci..

[37] Ferrari A.C., Robertson J. (2000). Interpretation of Raman spectra of disordered and amorphous carbon. Phys. Rev. B.

[38] Deng X., Gao S., Liu Y., Bao Y., Zhu Y., Fu Y. (2022). Cellular-like sericin-derived carbon decorated reduced graphene oxide for tunable microwave absorption. Appl. Surf. Sci..

[39] Cui J., Wang X., Huang L., Zhang C., Yuan Y., Li Y. (2022). Environmentally friendly bark-derived Co-Doped porous carbon composites for microwave absorption. Carbon.

[40] Cai Y., Cheng Y., Wang Z., Fei G., Lavorgna M., Xia H. (2023). Facile and scalable preparation of ultralight cobalt@graphene aerogel microspheres with strong and wide bandwidth microwave absorption. Chem. Eng. J..

[41] Wang B., Ji Y., Mu C., Huo Y., Xiang J., Nie A., Xue T., Zhai K., Liu Z., Wen F. (2022). Well-controlled Core-shell structures based on Fe_3_O_4_ nanospheres coated by polyaniline for highly efficient microwave absorption. Appl. Surf. Sci..

[42] Wang Q., Wu X., Huang J., Chen S., Zhang Y., Dong C., Chen G., Wang L., Guan H. (2021). Enhanced microwave absorption of biomass carbon/nickel/polypyrrole (C/Ni/PPy) ternary composites through the synergistic effects. J. Alloys Compd..

[43] Meng Y., Liu Y., Yang C., Kong L.B. (2023). NiFe_2_O_4_/coal-based carbon composites with magnetic properties and microwave absorption capacity prepared through microwave radiation and hydrothermal reaction. Synth. Met..

[44] Li X., Zhou H., Zhang J., Zhang X., Li M., Zhang J., Darwish M.A., Zhou T., Sun S.-K., Xie L. (2024). Construction of shell-like carbon superstructures through anisotropically oriented self-assembly for distinct electromagnetic wave absorption. J. Mater. Chem. A.

