# Supplementary material for: Facile Fabrication of Co-Doped Porous Carbon from Coal Hydrogasification Semi-Coke for Efficient Microwave Absorption
Source: Molecules. 2024 Sep 29;29(19):4633. doi: 10.3390/molecules29194633 (PMC11478072; doi:10.3390/molecules29194633)
Supplement: Supplementary file 1 [file molecules-29-04633-s001.zip › molecules-3219602-supplementary.pdf]

Facile Fabrication of Co-doped Porous Carbon from Coal Hydrogasification Semi-Coke for Efficient Microwave Absorption

Yan-Fang Li [1,2,*], Li-Fang Wang [1], Shu-Juan Gao [1,2], Tan-Lai Yu [1,2], Qi-Feng Li [3] and Jun-Wei Wang [3,*]

1 Department of Chemical and Materials Engineering, Lyuliang University, Lvliang 033001, China; lllswlf@163.com (L.-F.W.); shujuangao@llu.edu.cn (S.-J.G.); 20171017@llu.edu.cn (T.-L.Y.)
2 Institute of New Carbon-Based Materials and Zero-carbon and Negative-carbon Technology, Lyuliang University, Lvliang 033001, China
3 Institute of Coal Chemistry, Chinese Academy of Sciences, Taiyuan 033000, China; liqf@sxicc.ac.cn
* Correspondence: liyanfang@llu.edu.cn (Y.-F.L.); wangjw@sxicc.ac.cn (J.-W.W.)

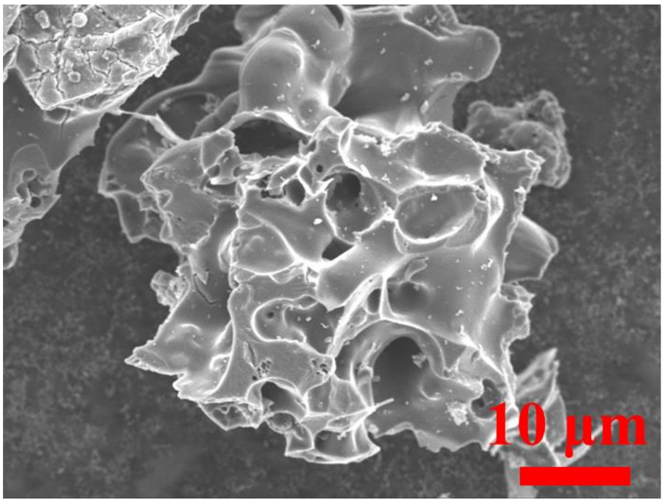

Figure S1. The SEM images of SC.

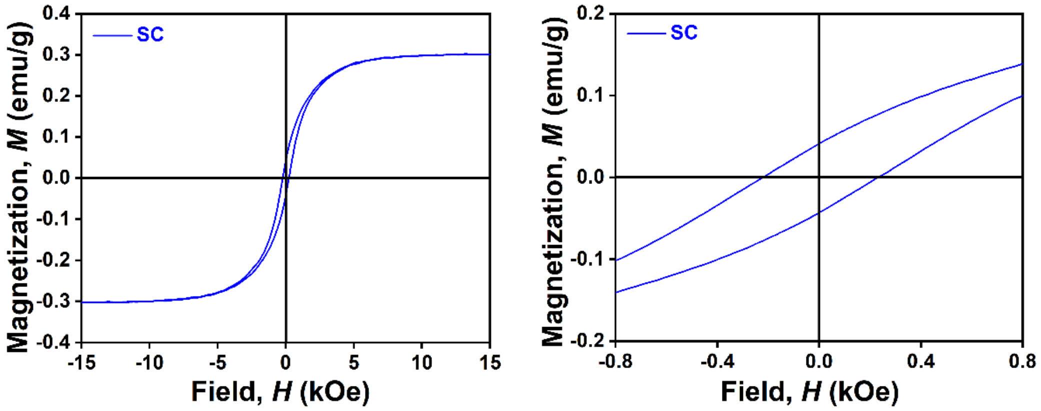

Figure S2. Magnetic hysteresis loops of SC.
